# Di­propyl­ammonium 4-amino­benzene­sulfonate

**DOI:** 10.1107/S2414314620006598

**Published:** 2020-05-29

**Authors:** Bougar Sarr, Abdou Mbaye, Assane Touré, Cheikh Abdoul Khadir Diop, Mamadou Sidibé, François Michaud

**Affiliations:** a Laboratoire de Chimie Minérale et Analytique, Département de Chimie, Faculté des Sciences et Téchniques, Université Cheikh Anta Diop, Dakar, Senegal; b Laboratoire de Chimie et de Physique des Matériaux (LCPM) de l’Université Assane, Seck de Ziguinchor (UASZ), BP 523 Ziguinchor, Senegal; c Service Commun d’Analyse par Diffraction des Rayons X, Université de Bretagne Occidentale, 6, avenue Victor Le Gorgeu, CS 93837, F-29238 BREST cedex 3, France; University of Aberdeen, Scotland

**Keywords:** crystal structure, hydrogen bonds, sulfanilate

## Abstract

In the title mol­ecular salt, NH_2_(C_3_H_7_)_2_^+^·[NH_2_C_6_H_4_SO_3_]^−^, the cation displays an extended conformation. In the crystal, anion-to-anion N—H⋯O and N—H⋯(O,O) hydrogen bonds generate (101) layers. Cation-to-anion N—H⋯O hydrogen bonds connect the layers into a three-dimensional network.

## Abstract 2

In the title mol­ecular salt, N–H⋯O and N–H⋯(O,*O*) hydrogen bonds connect the components into a three-dimensional network.

## Graphical abstract

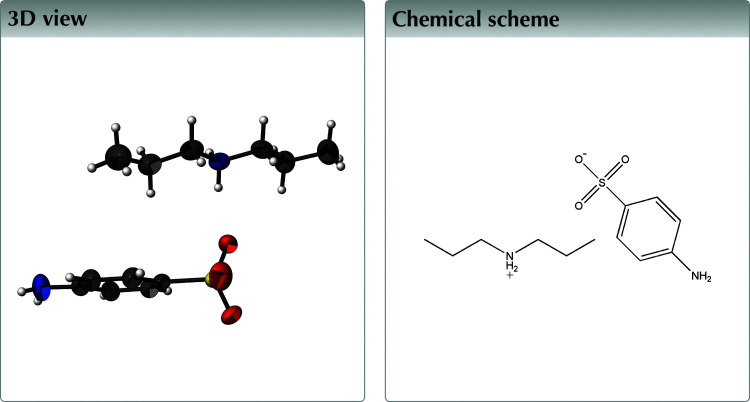

## Structure description

Some sulfanilate-based compounds have high optical non-linearity and may be candidates for applications in optoelectronics and photonics in combination with organic cations such as guanidinium (Russell *et al.*, 1994 ▸), tri­ethyl­ammonium (Li *et al.*, 2007 ▸), diiso­propyl­ammonium (Sarr *et al.*, 2016 ▸) and cyclo­hexyl­ammonium (Kama *et al.*, 2019 ▸). As part of our ongoing studies in this area (Sarr *et al.*, 2016 ▸), we now describe the synthesis and crystal structure of the title mol­ecular salt, which crystallizes in the non-centrosymmetric space group *Pn*.

**Figure 1:**
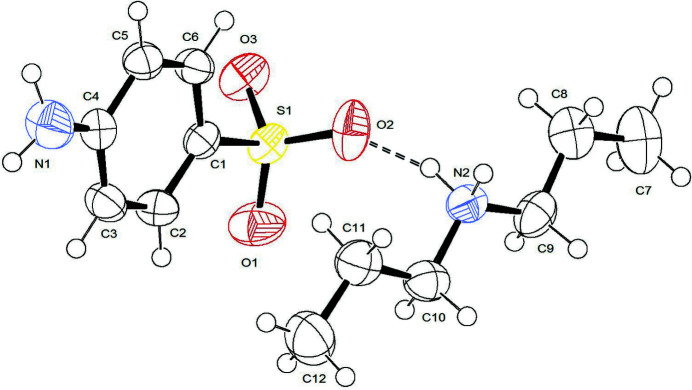
The asymmetric unit with displacement ellipsoids drawn at the 50% probability level. The N2—H2*B*⋯O2 hydrogen bond is shown as a dashed line.

The asymmetric unit, shown in Fig. 1 ▸, consists of one di­propyl­ammonium NH_2_(C_3_H_7_)_2_^+^ cation and one 4-amino­benzene­sulfonate [NH_2_C_6_H_4_SO_3_]^−^ anion. The cation adopts an extended structure with a minimum torsion angle of 174.7 (4)° for N2—C10—C11—C12. The involvement of the oxygen atoms of the sulfonate group in the anion as hydrogen-bond acceptors is manifested in a slight difference in the S—O bond lengths [S1—O1 = 1.446 (2), S1—O2 = 1.454 (2), S1—O3 = 1.449 (2) Å]: these data are consistent with those in sulfanilate anions previously reported (Sarr *et al.*, 2016 ▸; Kama *et al.*, 2019 ▸).

**Figure 2:**
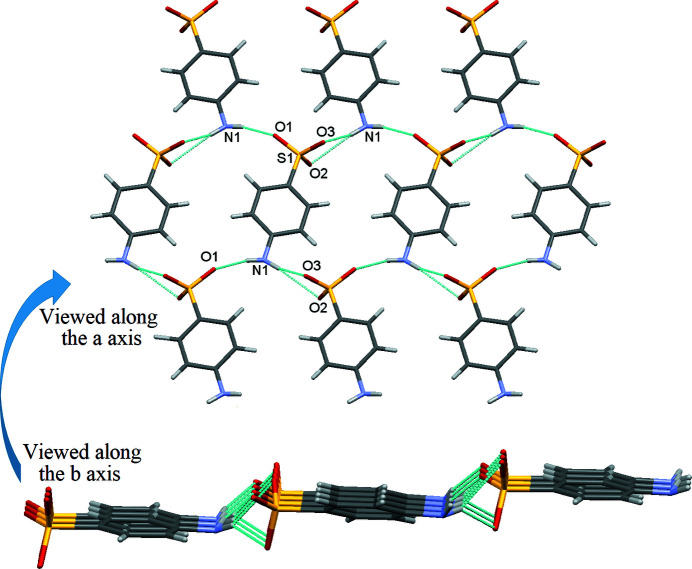
A perspective view of an infinite layer, viewed along the *a* and *b* axes.

**Figure 3:**
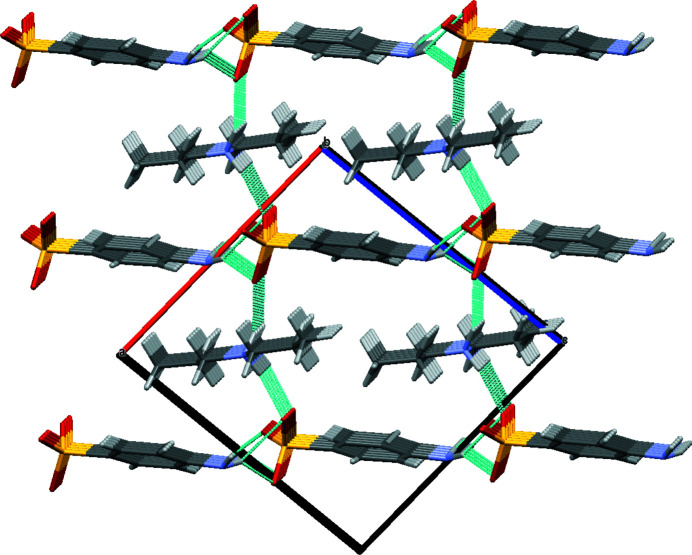
The packing viewed along [010].

In the extended structure, each sulfanilate anion inter­acts with four neighbours *via* simple N—H⋯O and bifurcated N—H⋯(O,O) hydrogen bonds (Table 1 ▸) to generate (101) layers (Fig. 2 ▸). The di­propyl­ammonium cations play the role of bridges between the infinite anion layers *via* cation-to-anion N—H⋯O hydrogen bonds (Fig. 3 ▸) to generate a three-dimensional network. Each sulfanilate anion is thus surrounded by four anions and two cations.

## Synthesis and crystallization

A 1:2 mixture of sulfanilic acid (1.00 g, 5.80 mmol) and di­propyl­ammine (1.16 g, 11.50 mmol) was dissolved in water and the colourless solution obtained was stirred for an hour. After a few days of evaporation in an oven at 333 K, some yellowish crystals were collected from the solution and then dried in air. The IR spectrum and peak assignments are given in the supporting information.

## Refinement

Crystal data, data collection and structure refinement details are summarized in Table 2 ▸.

## Supplementary Material

Crystal structure: contains datablock(s) global, I. DOI: 10.1107/S2414314620006598/hb4347sup1.cif

IR spectrum. DOI: 10.1107/S2414314620006598/hb4347sup3.docx

Supporting information file. DOI: 10.1107/S2414314620006598/hb4347Isup3.cml

CCDC reference: 2004571

Additional supporting information:  crystallographic information; 3D view; checkCIF report

## Figures and Tables

**Table 1:** Hydrogen-bond geometry (Å, °)

*D*—H⋯*A*	*D*—H	H⋯*A*	*D*⋯*A*	*D*—H⋯*A*
N1—H1*A*⋯O2^i^	0.90 (2)	2.39 (3)	3.196 (4)	149 (4)
N1—H1*A*⋯O3^i^	0.90 (2)	2.49 (3)	3.313 (4)	153 (4)
N1—H1*B*⋯O1^ii^	0.88 (2)	2.08 (2)	2.940 (4)	165 (4)
N2—H2*A*⋯O3^iii^	0.91 (2)	1.91 (2)	2.801 (3)	164 (3)
N2—H2*B*⋯O2	0.90 (2)	1.95 (3)	2.786 (3)	154 (3)

Symmetry codes: (i) 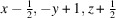 ; (ii) 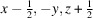 ; (iii) 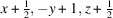 .

**Table 2:** Experimental details

Crystal data	
Chemical formula	C_6_H_16_N^+^·C_6_H_6_NO_3_S^−^
*M*_r_	274.38
Crystal system, space group	Monoclinic, *P* *n*
Temperature (K)	293
*a*, *b*, *c* (Å)	10.2564 (7), 6.5369 (5), 10.9683 (9)
β (°)	95.067 (7)
*V* (Å^3^)	732.50 (10)
*Z*	2
Radiation type	Mo *K*α
μ (mm^−1^)	0.22
Crystal size (mm)	0.38 × 0.28 × 0.19

Data collection	
Diffractometer	Agilent Xcalibur, Sapphire2
Absorption correction	Multi-scan (*SADABS*; Bruker, 2008 ▸)
*T*_min_, *T*_max_	0.669, 0.746
No. of measured, independent and observed [*I* > 2σ(*I*)] reflections	21351, 4018, 3387
*R*_int_	0.149
(sin θ/λ)_max_ (Å^−1^)	0.699

Refinement	
*R*[*F* ^2^ > 2σ(*F* ^2^)], *wR*(*F* ^2^), *S*	0.039, 0.097, 1.17
No. of reflections	4018
No. of parameters	181
No. of restraints	13
H-atom treatment	H atoms treated by a mixture of independent and constrained refinement
Δρ_max_, Δρ_min_ (e Å^−3^)	0.21, −0.28
Absolute structure	Flack (1983 ▸)
Absolute structure parameter	0.09 (6)

Computer programs: *SHELXT* (Sheldrick, 2015 ▸), *SHELXL97* (Sheldrick, 2008 ▸), *OLEX2* (Dolomanov *et al.*, 2009 ▸) *ORTEP-3 for Windows* (Farrugia, 2012 ▸) *Mercury* (Macrae *et al.*, 2020 ▸), *OLEX2* (Dolomanov *et al.*, 2009 ▸), *WinGX* (Farrugia, 2012 ▸), *PLATON* (Spek, 2020 ▸), *enCIFer* (Allen *et al.*, 2004 ▸).

## full crystallographic data

### Crystal data

**Table:**

C_6_H_16_N^+^·C_6_H_6_NO_3_S^−^	*F*(000) = 296
*M**_r_* = 274.38	*D*_x_ = 1.244 Mg m^−^^3^
Monoclinic, *P**n*	Mo *K*α radiation, λ = 0.71073 Å
*a* = 10.2564 (7) Å	Cell parameters from 38522 reflections
*b* = 6.5369 (5) Å	θ = 3.5–37.4°
*c* = 10.9683 (9) Å	µ = 0.22 mm^−^^1^
β = 95.067 (7)°	*T* = 293 K
*V* = 732.50 (10) Å^3^	Block, clear light colourless
*Z* = 2	0.38 × 0.28 × 0.19 mm

### Data collection

**Table:**

Agilent Xcalibur, Sapphire2 diffractometer	3387 reflections with *I* > 2σ(*I*)
Graphite monochromator	*R*_int_ = 0.149
ω scans	θ_max_ = 29.8°, θ_min_ = 6.4°
Absorption correction: multi-scan (SADABS; Bruker, 2008)	*h* = −13→14
*T*_min_ = 0.669, *T*_max_ = 0.746	*k* = −9→9
21351 measured reflections	*l* = −15→15
4018 independent reflections	

### Refinement

**Table:**

Refinement on *F*^2^	Secondary atom site location: difference Fourier map
Least-squares matrix: full	Hydrogen site location: inferred from neighbouring sites
*R*[*F*^2^ > 2σ(*F*^2^)] = 0.039	H atoms treated by a mixture of independent and constrained refinement
*wR*(*F*^2^) = 0.097	*w* = 1/[σ^2^(*F*_o_^2^) + (0.*P*)^2^ + 0.0621*P*] where *P* = (*F*_o_^2^ + 2*F*_c_^2^)/3
*S* = 1.17	(Δ/σ)_max_ = 0.003
4018 reflections	Δρ_max_ = 0.21 e Å^−^^3^
181 parameters	Δρ_min_ = −0.28 e Å^−^^3^
13 restraints	Absolute structure: Flack (1983)
34 constraints	Absolute structure parameter: 0.09 (6)
Primary atom site location: structure-invariant direct methods	

### Special details

**Table:**

Geometry. All esds (except the esd in the dihedral angle between two l.s. planes) are estimated using the full covariance matrix. The cell esds are taken into account individually in the estimation of esds in distances, angles and torsion angles; correlations between esds in cell parameters are only used when they are defined by crystal symmetry. An approximate (isotropic) treatment of cell esds is used for estimating esds involving l.s. planes.
Refinement. Refinement of F^2^ against ALL reflections. The weighted R-factor wR and goodness of fit S are based on F^2^, conventional R-factors R are based on F, with F set to zero for negative F^2^. The threshold expression of F^2^ > 2σ(F^2^) is used only for calculating R-factors(gt) etc. and is not relevant to the choice of reflections for refinement. R-factors based on F^2^ are statistically about twice as large as those based on F, and R- factors based on ALL data will be even larger.The C-bound H atoms were geometrically placed and refined as riding atoms. The N-bound H atoms were located in difference maps and their positions were freely refined.

### Fractional atomic coordinates and isotropic or equivalent isotropic displacement parameters (Å^2^)

**Table:**

	*x*	*y*	*z*	*U*_iso_*/*U*_eq_	
C1	0.2799 (2)	0.3112 (4)	0.2052 (2)	0.0367 (5)	
C2	0.2417 (3)	0.1097 (4)	0.2172 (3)	0.0445 (6)	
H2	0.265741	0.011733	0.161908	0.053*	
C3	0.1673 (3)	0.0538 (4)	0.3118 (3)	0.0478 (6)	
H3	0.142230	−0.082099	0.319082	0.057*	
C4	0.1296 (3)	0.1968 (4)	0.3958 (2)	0.0422 (6)	
C5	0.1670 (3)	0.4010 (4)	0.3810 (3)	0.0437 (6)	
H5	0.141106	0.500353	0.434520	0.052*	
C6	0.2419 (3)	0.4561 (4)	0.2879 (2)	0.0413 (6)	
H6	0.267171	0.591786	0.280253	0.050*	
N1	0.0600 (3)	0.1413 (5)	0.4919 (3)	0.0648 (8)	
O1	0.3872 (3)	0.2145 (4)	0.0083 (2)	0.0685 (7)	
O2	0.4990 (2)	0.4606 (4)	0.1461 (2)	0.0629 (6)	
O3	0.3054 (2)	0.5583 (3)	0.02517 (18)	0.0554 (6)	
H1A	0.012 (4)	0.243 (5)	0.520 (4)	0.088 (14)*	
H1B	0.018 (3)	0.024 (5)	0.488 (4)	0.072 (12)*	
C7	0.9403 (4)	0.6507 (8)	0.1124 (4)	0.0829 (12)	
H7A	0.909563	0.612015	0.030421	0.124*	
H7B	1.021641	0.582605	0.135713	0.124*	
H7C	0.953392	0.796103	0.116045	0.124*	
C8	0.8400 (3)	0.5899 (5)	0.1991 (3)	0.0614 (9)	
H8A	0.867832	0.640996	0.280284	0.074*	
H8B	0.757055	0.654361	0.172644	0.074*	
C9	0.8201 (3)	0.3642 (5)	0.2060 (3)	0.0530 (7)	
H9A	0.901763	0.299306	0.235920	0.064*	
H9B	0.794755	0.311445	0.124654	0.064*	
C10	0.6895 (3)	0.0913 (5)	0.3018 (3)	0.0516 (8)	
H10A	0.768416	0.021660	0.334665	0.062*	
H10B	0.663027	0.033385	0.221981	0.062*	
C11	0.5834 (4)	0.0585 (5)	0.3850 (3)	0.0591 (8)	
H11A	0.507946	0.140033	0.355995	0.071*	
H11B	0.613660	0.106484	0.466270	0.071*	
C12	0.5419 (4)	−0.1628 (7)	0.3930 (5)	0.0802 (12)	
H12A	0.512348	−0.211830	0.312830	0.120*	
H12B	0.472165	−0.173525	0.445549	0.120*	
H12C	0.614985	−0.243529	0.425863	0.120*	
N2	0.7169 (2)	0.3125 (4)	0.2888 (2)	0.0435 (5)	
S1	0.37479 (6)	0.38949 (10)	0.08711 (6)	0.04080 (17)	
H2A	0.734 (3)	0.372 (4)	0.364 (2)	0.064 (10)*	
H2B	0.644 (2)	0.382 (4)	0.264 (3)	0.069 (12)*	

### Atomic displacement parameters (Å^2^)

**Table:**

	*U*^11^	*U*^22^	*U*^33^	*U*^12^	*U*^13^	*U*^23^
C1	0.0348 (12)	0.0419 (13)	0.0332 (11)	0.0043 (10)	0.0018 (9)	0.0014 (9)
C2	0.0517 (17)	0.0382 (13)	0.0438 (14)	0.0021 (11)	0.0060 (12)	−0.0044 (11)
C3	0.0528 (16)	0.0376 (13)	0.0533 (16)	−0.0064 (12)	0.0073 (13)	−0.0001 (12)
C4	0.0381 (13)	0.0457 (14)	0.0427 (13)	−0.0032 (11)	0.0033 (11)	0.0007 (11)
C5	0.0461 (15)	0.0431 (14)	0.0427 (14)	−0.0006 (11)	0.0087 (12)	−0.0080 (10)
C6	0.0455 (14)	0.0349 (12)	0.0437 (14)	−0.0006 (10)	0.0049 (12)	−0.0033 (10)
N1	0.072 (2)	0.0622 (18)	0.0641 (18)	−0.0099 (15)	0.0292 (16)	0.0007 (14)
O1	0.0877 (17)	0.0627 (14)	0.0594 (13)	0.0110 (13)	0.0303 (12)	−0.0067 (12)
O2	0.0394 (11)	0.0936 (17)	0.0546 (12)	−0.0071 (11)	−0.0020 (10)	0.0245 (12)
O3	0.0606 (13)	0.0637 (13)	0.0416 (11)	0.0157 (11)	0.0023 (10)	0.0158 (10)
C7	0.066 (2)	0.116 (3)	0.065 (2)	−0.023 (2)	0.0032 (19)	0.010 (2)
C8	0.061 (2)	0.073 (2)	0.0502 (17)	−0.0077 (17)	0.0041 (16)	0.0003 (15)
C9	0.0443 (16)	0.069 (2)	0.0448 (15)	0.0084 (14)	0.0000 (13)	−0.0059 (13)
C10	0.058 (2)	0.0487 (17)	0.0464 (16)	0.0045 (13)	−0.0052 (14)	−0.0062 (12)
C11	0.062 (2)	0.0575 (19)	0.0567 (18)	0.0025 (16)	−0.0017 (16)	0.0008 (15)
C12	0.075 (3)	0.068 (2)	0.096 (3)	−0.010 (2)	0.000 (2)	0.012 (2)
N2	0.0454 (12)	0.0454 (13)	0.0383 (11)	0.0061 (11)	−0.0038 (9)	−0.0062 (10)
S1	0.0402 (3)	0.0483 (3)	0.0339 (3)	0.0074 (3)	0.0033 (2)	0.0044 (3)

### Geometric parameters (Å, º)

**Table:**

C1—C2	1.384 (3)	C7—C8	1.515 (5)
C1—C6	1.391 (3)	C8—H8A	0.9700
C1—S1	1.763 (3)	C8—H8B	0.9700
C2—H2	0.9300	C8—C9	1.492 (5)
C2—C3	1.390 (4)	C9—H9A	0.9700
C3—H3	0.9300	C9—H9B	0.9700
C3—C4	1.391 (4)	C9—N2	1.493 (4)
C4—C5	1.402 (4)	C10—H10A	0.9700
C4—N1	1.373 (4)	C10—H10B	0.9700
C5—H5	0.9300	C10—C11	1.496 (5)
C5—C6	1.378 (4)	C10—N2	1.483 (4)
C6—H6	0.9300	C11—H11A	0.9700
N1—H1A	0.90 (2)	C11—H11B	0.9700
N1—H1B	0.88 (2)	C11—C12	1.513 (5)
O1—S1	1.446 (2)	C12—H12A	0.9600
O2—S1	1.454 (2)	C12—H12B	0.9600
O3—S1	1.449 (2)	C12—H12C	0.9600
C7—H7A	0.9600	N2—H2A	0.91 (2)
C7—H7B	0.9600	N2—H2B	0.90 (2)
C7—H7C	0.9600		
			
C2—C1—C6	119.2 (2)	C8—C9—N2	111.2 (2)
C2—C1—S1	121.7 (2)	H9A—C9—H9B	108.0
C6—C1—S1	119.1 (2)	N2—C9—H9A	109.4
C1—C2—H2	120.0	N2—C9—H9B	109.4
C1—C2—C3	120.0 (3)	H10A—C10—H10B	108.1
C3—C2—H2	120.0	C11—C10—H10A	109.5
C2—C3—H3	119.3	C11—C10—H10B	109.5
C2—C3—C4	121.4 (3)	N2—C10—H10A	109.5
C4—C3—H3	119.3	N2—C10—H10B	109.5
C3—C4—C5	117.9 (2)	N2—C10—C11	110.7 (3)
N1—C4—C3	121.6 (3)	C10—C11—H11A	108.9
N1—C4—C5	120.4 (3)	C10—C11—H11B	108.9
C4—C5—H5	119.6	C10—C11—C12	113.3 (3)
C6—C5—C4	120.7 (2)	H11A—C11—H11B	107.7
C6—C5—H5	119.6	C12—C11—H11A	108.9
C1—C6—H6	119.6	C12—C11—H11B	108.9
C5—C6—C1	120.8 (2)	C11—C12—H12A	109.5
C5—C6—H6	119.6	C11—C12—H12B	109.5
C4—N1—H1A	114 (3)	C11—C12—H12C	109.5
C4—N1—H1B	118 (3)	H12A—C12—H12B	109.5
H1A—N1—H1B	113 (4)	H12A—C12—H12C	109.5
H7A—C7—H7B	109.5	H12B—C12—H12C	109.5
H7A—C7—H7C	109.5	C9—N2—H2A	112 (2)
H7B—C7—H7C	109.5	C9—N2—H2B	109 (2)
C8—C7—H7A	109.5	C10—N2—C9	115.5 (2)
C8—C7—H7B	109.5	C10—N2—H2A	110.4 (19)
C8—C7—H7C	109.5	C10—N2—H2B	111 (2)
C7—C8—H8A	108.9	H2A—N2—H2B	98 (3)
C7—C8—H8B	108.9	O1—S1—C1	107.05 (13)
H8A—C8—H8B	107.7	O1—S1—O2	113.52 (17)
C9—C8—C7	113.2 (3)	O1—S1—O3	112.81 (14)
C9—C8—H8A	108.9	O2—S1—C1	106.52 (12)
C9—C8—H8B	108.9	O3—S1—C1	106.58 (12)
C8—C9—H9A	109.4	O3—S1—O2	109.88 (15)
C8—C9—H9B	109.4		
			
C1—C2—C3—C4	0.1 (4)	C6—C1—S1—O1	−173.8 (2)
C2—C1—C6—C5	0.1 (4)	C6—C1—S1—O2	64.5 (2)
C2—C1—S1—O1	5.8 (3)	C6—C1—S1—O3	−52.8 (2)
C2—C1—S1—O2	−116.0 (2)	N1—C4—C5—C6	177.1 (3)
C2—C1—S1—O3	126.7 (2)	C7—C8—C9—N2	−177.9 (3)
C2—C3—C4—C5	1.1 (4)	C8—C9—N2—C10	179.9 (3)
C2—C3—C4—N1	−177.7 (3)	C11—C10—N2—C9	−179.1 (3)
C3—C4—C5—C6	−1.7 (4)	N2—C10—C11—C12	174.7 (3)
C4—C5—C6—C1	1.2 (4)	S1—C1—C2—C3	179.8 (2)
C6—C1—C2—C3	−0.7 (4)	S1—C1—C6—C5	179.6 (2)

### Hydrogen-bond geometry (Å, º)

**Table:**

*D*—H···*A*	*D*—H	H···*A*	*D*···*A*	*D*—H···*A*
N1—H1*A*···O2^i^	0.90 (2)	2.39 (3)	3.196 (4)	149 (4)
N1—H1*A*···O3^i^	0.90 (2)	2.49 (3)	3.313 (4)	153 (4)
N1—H1*B*···O1^ii^	0.88 (2)	2.08 (2)	2.940 (4)	165 (4)
N2—H2*A*···O3^iii^	0.91 (2)	1.91 (2)	2.801 (3)	164 (3)
N2—H2*B*···O2	0.90 (2)	1.95 (3)	2.786 (3)	154 (3)

Symmetry codes: (i) *x*−1/2, −*y*+1, *z*+1/2; (ii) *x*−1/2, −*y*, *z*+1/2; (iii) *x*+1/2, −*y*+1, *z*+1/2.
