# Supplementary figures and images for: Di­propyl­ammonium 4-amino­benzene­sulfonate
Source: IUCrdata. 2020 May 29;5(Pt 5):x200659. doi: 10.1107/S2414314620006598 (PMC9462226; doi:10.1107/S2414314620006598)

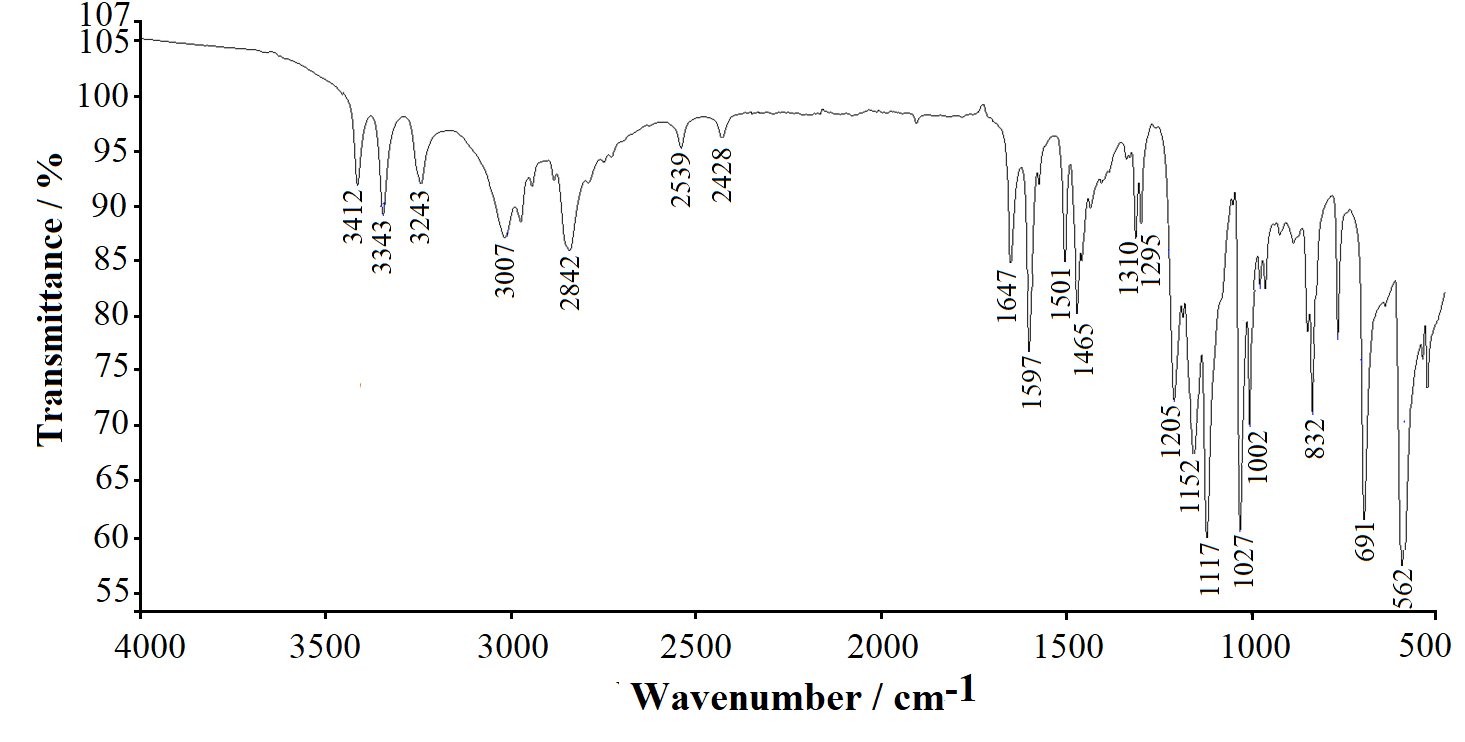


Fig S1. IR spectrum of (I)

Supplement: Supplementary file 2 [file x-05-x200659-sup3.docx]
